# Short-Term Medical Cannabis Treatment Regimens Produced Beneficial Effects among Palliative Cancer Patients

**DOI:** 10.3390/ph13120435

**Published:** 2020-11-30

**Authors:** Joshua Aviram, Gil M. Lewitus, Yelena Vysotski, Anton Uribayev, Shiri Procaccia, Idan Cohen, Anca Leibovici, Mahmud Abo-Amna, Luiza Akria, Dmitry Goncharov, Neomi Mativ, Avia Kauffman, Ayelet Shai, Or Hazan, Gil Bar-Sela, David Meiri

**Affiliations:** 1 Faculty of Biology, Technion-Israel Institute of Technology, Haifa 32000, Israel; shukiaviram@gmail.com (J.A.); lgil@technion.ac.il (G.M.L.); lalatrololo@gmail.com (Y.V.); shiri.procaccia@gmail.com (S.P.); orhazan@gmail.com (O.H.); 2 Department of Oncology, Galilee Medical Center, Nahariya 22100, Israel; AntonU@gmc.gov.il (A.U.); AncaL@gmc.gov.il (A.L.); AkriaL@gmc.gov.il (L.A.); DmitriG@gmc.gov.il (D.G.); NeomyM@gmc.gov.il (N.M.); AviaK@gmc.gov.il (A.K.); ayelets@gmc.gov.il (A.S.); 3 Cancer Center, Emek Medical Center, Afula 18101, Israel; idan5161@gmail.com (I.C.); mahmud_ab@clalit.org.il (M.A.-A.); 4 Faculty of Medicine, Technion-Israel Institute of Technology, Haifa 32000, Israel

**Keywords:** medical cannabis, THC, CBD, palliative cancer treatment, oncology

## Abstract

In the last decade the use of medical cannabis (MC) for palliative cancer treatment has risen. However, the choice between products is arbitrary and most patients are using Tetrahydrocannabinol (THC)-dominant cannabis products. In this study, we aimed to assess the short-term outcomes of MC treatment prescribed by oncologists in relation to the type of cannabis they receive. A comparative analysis was used to assess the differences in treatment effectiveness and safety between THC-dominant (*n* = 56, 52%), cannabidiol (CBD)-dominant (*n* = 19, 18%), and mixed (*n* = 33, 30%) MC treatments. Oncology patients (*n* = 108) reported on multiple symptoms in baseline questionnaires, initiated MC treatment, and completed a one-month follow-up. Most parameters improved significantly from baseline, including pain intensity, affective and sensory pain, sleep quality and duration, cancer distress, and both physical and psychological symptom burden. There was no significant difference between the three MC treatments in the MC-related safety profile. Generally, there were no differences between the three MC treatments in pain intensity and in most secondary outcomes. Unexpectedly, CBD-dominant oil treatments were similar to THC-dominant treatments in their beneficial effects for most secondary outcomes. THC-dominant treatments showed significant superiority in their beneficial effect only in sleep duration compared to CBD-dominant treatments. This work provides evidence that, though patients usually consume THC-dominant products, caregivers should also consider CBD-dominant products as a useful treatment for cancer-related symptoms.

## 1. Introduction

Cancer patients suffer from many conditions resulting from the disease or its treatment. These include cancer-related pain [1], anxiety [2], depression [3], insomnia [4], decreased quality of life [5], and increased disability [6]. These comorbidities are some of the underlying causes of cancer patients’ suffering while undergoing therapies and some may even lead to worsened prognosis [3]. To date, there is still no optimal treatment addressing all of these comorbidities [7].

Currently, Medical Cannabis (MC) is one of the options to alleviate cancer patients’ suffering [8]. While the preclinical literature is vast [9,10,11,12,13], there is a paucity of clinical literature [14], leading to arbitrary MC treatment regimen decisions based mostly on the oncologist experience and on patient demands. Many oncologists find MC appropriate as the first-line therapy for cancer-related symptoms [15] and a recent study showed that the majority of cancer patients request MC treatment from their oncologist [16]. Nonetheless, there is not enough data on the negative and positive effects of MC treatment on cancer patients [17].

Importantly, cannabis is not a single compound; it is comprised from many compounds in different concentrations, including many phytocannabinoids [18,19] with diverse biological activities [20,21]. The concentration of these compounds (i.e., chemovar) is also vastly different between different strains (i.e., cultivars) [22,23,24]. This makes traditional treatment by titrating of a single molecule particularly difficult. Thus far, Randomized Controlled Trials of cancer-related pain focused mainly on (-)-Δ^9^-*trans*-tetrahydrocannabinol (THC) [25,26,27,28,29,30,31], but currently, probably due to the large media hype and its more enabling regulation worldwide, there is a major shift of focus to cannabidiol (CBD) [32]. Today, most countries that approve MC treatment require cultivators to report and adhere only by the two major phytocannabinoids, THC and CBD [33].

In this study, we conducted a prospective, short-term study that compared the effectiveness and safety of the most widely used categorization of MC treatment, conceptualized by Small et al., (1973), to THC-dominant as type I cannabis, equal THC and CBD as type II, and CBD-dominant as type III [34].

## 2. Results

**Figure 1:**
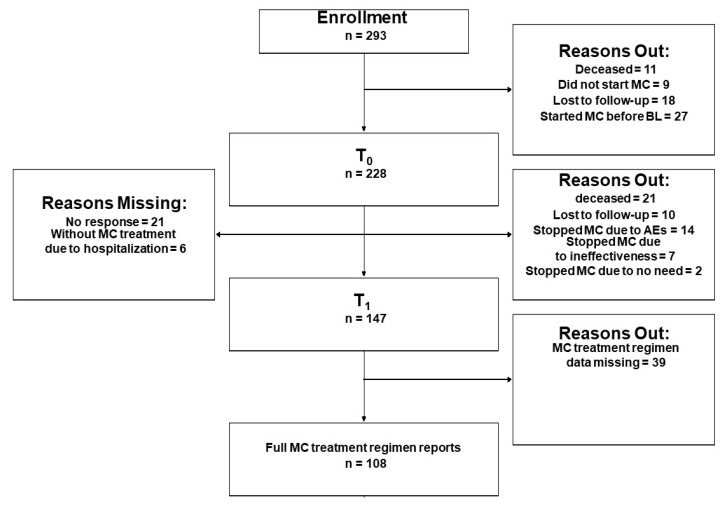
Consort flowchart diagram. T_0_, Baseline; T_1_, one-month follow-up; MC, medical cannabis; BL, baseline; AEs, adverse effects.

A total of 293 patients were enrolled to the study, of them, 228 patients completed the baseline (T_0_) questionnaires and initiated MC treatment (Figure 1, CONSORT flow diagram). A follow-up questionnaire was completed by 147 patients at T_1_. Of these patients, 108 patients reported on full MC treatment information, these patients represent the analyzed sample for the current study. About 95% of the patients provided data online and the rest by telephone calls.

### 2.1. MC Treatment Characteristics

MC treatment information was reported by 108 patients at T_1_ and included type I (*n* = 56, 52%; patients consuming THC-dominant cultivars only; included three products: THC(T)20/CBD(C)4, T15/C3 and T10/C2), type III (*n* = 19, 18%; patients consuming CBD-dominant cultivars only; included three products: T1/C20, T5/C10 and T3/C15), and type II (i.e., mixed; *n* = 33, 30%; patients consuming cultivars with similar THC:CBD ratio that included two products: T10/C10 and T5/C5, or patients consuming both THC-dominant and CBD-dominant cultivars in the same day) MC treatments. Sublingual MC oil extract was the most common route of administration for type III and for type II treatments and less common for type I treatments (*n* = 17, 89%, *n* = 19, 58%, and *n* = 19, 34%, respectively), whereas patients consuming type I treatments consumed it mostly (*n* = 32, 57%) by inflorescence inhalation. Inflorescence inhalation was less common for type III and for type II treatments (*n* = 2, 11% and *n* = 7, 21%, respectively). Some patients consuming type I and type II (*n* = 5, 9% and *n* = 7, 21%), but not for type III, utilized both administration routes (χ^2^_(2)_ = 26.02, *p* < 0.001). Overall reported monthly MC dose was similar for all three treatments (20 (20–20) grams).

THC and CBD monthly doses were significantly different between the three treatments (χ^2^_(2)_ = 58.07, *p* < 0.001 and χ^2^_(2)_ = 68.28, *p* < 0.001, respectively). Patients consuming type I treatments consumed 600 (400–725) mg/month of CBD (with weight-adjusted dose of 7.8 (5.7–11) mg/kg/month) and 3000 (2000–3600) mg/month of THC (with weight-adjusted dose of 39 (29–56) mg/kg/month). Patients consuming type III treatments consumed 2000 (2000–3000) mg/month of CBD (with weight-adjusted dose of 40 (26–46) mg/kg/month) and 1000 (600–1000) mg/month of THC (with weight-adjusted dose of 12 (8.4–15) mg/kg/month). Patients consuming type II treatments consumed 2000 (1500–2000) mg/month of CBD (with weight-adjusted dose of 30 (20–36) mg/kg/month) and 2000 (1400–2000) mg/month of THC (with weight-adjusted dose of 29 (20–36) mg/kg/month).

### 2.2. Baseline Demographics and Cancer Characteristics

Sixty-two of the patients in the sample were females (57%) with an average age of 64 (52–72) years. Demographic characteristics did not differ between the MC treatments (Table 1). Oncology diagnoses were heterogenous, with breast cancer being the most frequent diagnosis (*n* = 30, 28%), followed by lung, colon and ovarian cancers (*n* = 15, 14%, *n* = 15, 14%, and *n* = 5, 5%; respectively). Most patients (*n* = 48, 44%) were categorized as in IV stage cancer where 57 of the patients (53%) were at first-line of oncology treatment, meaning that most patients were diagnosed and started treatment while in advanced metastatic disease. Chemotherapy was the most prevalent current treatment protocol (*n* = 56, 52%), followed by biological and immunological cancer treatments protocols (*n* = 15, 14% and *n* = 10, 9%, respectively). Most patients (*n* = 76, 70%) were scored by the oncologist as not disabled (scored ≥ 1 based on Eastern Cooperative Oncology Group (ECOG) Performance Status score). Additionally, no significant differences were found in cancer characteristics between the three MC treatments (Table 2).

### 2.3. MC Treatment Safety

Fourteen patients stopped MC treatment due to Adverse Effects (AEs), such as dizziness (*n* = 2), hallucinations (*n* = 2), anxiety (*n* = 1), faints (*n* = 1), fatigue (*n* = 1), nausea (*n* = 1), combination of bad taste and drowsiness (*n* = 1), and combination of restlessness and weakness (*n* = 1); four patients did not specify the AEs that led to MC treatment discontinuation. Therefore, these patients are not analyzed in the T_1_ dataset.

Overall, 24 patients (22%) reported on at least one MC-related AE that did not led to MC treatment discontinuation. In a descending order, AEs consisted of central nervous system (CNS; *n* = 14, 13%), gastrointestinal (GI, *n* = 9, 8%), psychological (*n* = 7, 7%), musculoskeletal (*n* = 4, 4%), ophthalmic (*n* = 4, 4%), cardiovascular (*n* = 2, 2%), and auditory (*n* = 2, 2%) AEs. No significant differences were found between the three MC treatment regimens in MC-related AEs by affected systems (Table 3). 

The most frequent specific AEs were dizziness and tiredness (*n* = 9, 8%; for both) (Table 4). Due to the non-frequent reports of specific AEs, we could not assess the differences between them for the three MC treatments.

Notably, 21 (13%) patients that are not analyzed in the T_1_ dataset died during the first month of MC treatment. Therefore, their MC treatment type is unknown.

### 2.4. MC Treatment Regimens’ Effect

Assessing the effect of MC treatment (the change from T_0_ to T_1_), for all of the evaluated parameters, demonstrated that there was a significant (*p* < 0.05) improvement for several of the parameters. Specifically, significant improvement from baseline was reported for weekly average pain intensity, affective and sensory pain intensities, sleep quality and duration and in MSAS distress, physical (*p* < 0.01) and psychological indexes. Additionally, a decrease in analgesics consumption was also demonstrated. Notably, we found no significant change from T_0_ to T_1_ for least and worst pain intensities, weight, body mass index (BMI), pain catastrophizing scale, sleep latency, depression, disability, QoL, and for anxiety (*p* > 0.05) (Table 5).

**Figure 2:**
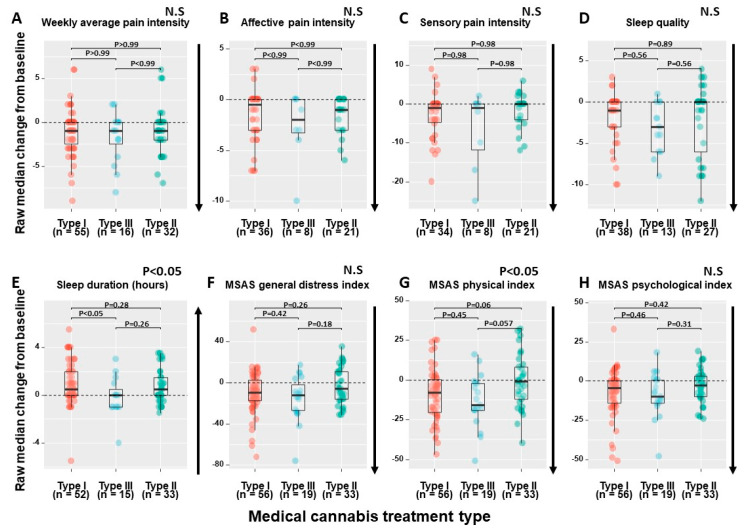
MC Treatment regimens significant differential effects. (**A**) Differences between the three MC treatments in weekly average pain intensity; (**B**) Differences between the three MC treatments in affective pain intensity; (**C**) Differences between the three MC treatments in sensory pain intensity; (**D**) Differences between the three MC treatments in sleep quality; (**E**) Differences between the three MC treatments in sleep duration; (**F**) Differences between the three MC treatments in MSAS general distress index; (**G**) Differences between the three MC treatments in MSAS physical index; (**H**) Differences between the three MC treatments in MSAS psychological index; CBD, Cannabidiol; THC, (−)-Δ^9^-*trans*-tetrahydrocannabinol; MSAS, Memorial Symptom Assessment Scale; The dashed lines represent the baseline (T_0_) values of their corresponding parameters; The box-plot values represent the raw median change from baseline (T_0_) to one-month follow-up (T_1_); *n*, Number of patients; N.S, Non-significant; Type I, THC-dominant treatments; Type III, CBD-dominant treatments; Type II, equal THC:CBD concentration treatments; Median and IQR change from baseline are calculated individually for each patient, the presented change from BL is the median of all individual patients and not the difference between the medians of BL and one-month follow-up; *p* values are adjusted for multiple comparisons; The direction of the arrows indicates the desired symptoms improvement trajectory.

For the above-mentioned parameters that improved significantly from T_0_ to T_1_, we analyzed the differences between the three MC treatments. We found that the median change from T_0_ to T_1_ between the three MC treatments was significantly different in MSAS physical index (χ^2^_(2)_ = 6.91, *p* < 0.05) and in sleep duration (χ^2^_(2)_ = 6.02, *p* < 0.05). Specifically, for MSAS physical index, post hoc analyses showed a trend for superiority of type III and type I treatments (−16 (−2 to 19) and −8 (+0.25 to −20)), respectively) compared to mixed treatments (−1 (+8 to −12)). Moreover, for MSAS physical index ≥30% clinical improvement, patients consuming type III treatments reported higher rate of response (*n* = 14, 74%), compared to type I (*n* = 28, 50%) and type II (*n* = 10, 30%) treatments (χ^2^_(2)_ = 9.24, *p* < 0.01). For sleep duration, post hoc analyses showed significance of superiority for type I treatments (+0.5 (0 to +2) h) compared to type III treatments (0 (−1 to +0.5) h) (Figure 2).

While there was a general decrease in analgesic medications consumption, there were no significant differences between the three MC treatments in the rates of patients that consumed them at T_0_ and stopped at T_1_ (χ^2^_(2)_ = 0.28, *p* = 0.87).

As patients can consume either sublingual oil extract or inflorescence by inhalation, we also compared between these different routes of administration. No significant differences were found between patients that consumed only type I cultivars by sublingual oil extract (*n* = 19) and patients that consumed only type I cultivars by inflorescence inhalation (*n* = 32) in the median change from T_0_ to T_1_ of all parameters (*p* > 0.05). In addition, comparative analysis between patients that consumed only type III cultivars by sublingual oil extract (*n* = 17) and patients that consumed only type I cultivars by inflorescence inhalation (*n* = 32) demonstrated that there was no significant difference in the median change from T_0_ to T_1_ of most parameters, excluding sleep duration. Overall, we found superiority for consumption of type I treatments by inflorescence inhalation (+1 (0.31 to +2.4) h) compared to consumption of type III treatments by sublingual oil extract in sleep duration extension (0 (−1 to +0.5) h) (χ^2^_(1)_ = 0.50, *p* < 0.05).

## 3. Discussion

In this “real-world” study on palliative care in oncology patients, we found a significant improvement in most of the assessed parameters, including reduced pain intensity, improved sleep, alleviated cancer symptoms and a decrease in pharmaceutical analgesics consumption. We demonstrated that this improvement from baseline is rapid and apparent, already at one month after MC initiation. This symptom relief profile is supported by previous studies [35,36,37,38,39,40], while it is opposite to the expected trajectory of symptoms in palliative cancer patients [41]. However, unlike previous studies that demonstrated an increase of appetite and weight gain [42,43], as well as anxiolytic [44], antidepressant [45], and quality of life (QoL) improvement properties [46]; we found no significant short-term improvement for those in the allocated time. These findings may be explained by the inherent nature of the parameters, they may require additional treatment duration for the effects to be apparent. Another possibility is the patients’ demographics, as these are palliative oncology patients with high rates of advanced metastatic disease. These results can assist oncologists to facilitate patients’ expectations regarding the short-term effects of MC treatment.

Additionally, patients reported a mostly non-serious adverse effects profile. Our findings are in line with previous results concluding that cannabinoid treatment for cancer-related pain is safe [47]. A previous study of recreational cannabis users demonstrated that THC is a major contributor to the psychoactive effects of cannabis, with dose-dependent properties [48], whereas CBD is generally described in psychiatric clinical studies as safe or as the phytocannabinoid that attenuates THCs’ psychoactive effects [49]. Moreover, the American Pain Society (APS) guidelines prefer to direct patients to low-THC and high-CBD content MC cultivars due to THC-related AEs [50]. In our study, we found no significant differences between MC treatment regimens in MC-related AEs. Thus, the dominance of THC/CBD phytocannabinoids within the MC treatments, at least for short-term treatment of palliative cancer patients, does not play a clinical role in the safety profile of MC.

Currently, oncologists in Israel are required to prescribe MC treatment deciding on the administration route and on THC:CBD ratio of products. We found that most of the prescriptions for palliative oncology patients were for type I cultivars, which might be explained by previous clinical trials demonstrating that cultivars with higher THC content provide a better therapeutic response for pain reduction [51]. In the current study we found that except for sleep duration, type III treatments were as good as type I treatments. In fact, for most assessed parameters, there was no superiority of any specific MC treatment. Additionally, a trend of significance was found for both type I and type III treatments compared to type II treatments for cancer-related physical symptoms reduction, with higher clinical response rate for type III treatments. Hence, we can assume, based on our results, that for amelioration of physical cancer symptoms, and for most cancer-related parameters (other than sleep duration extension), there is no added therapeutic value for type I treatments and physicians can prescribe type III cultivars. Additionally, Portenoy et al., (2012) showed that lower THC and CBD concentrations of Nabiximoles were associated with higher pain reduction efficacy in cancer patients [29]. Hence, the higher response rate for physical cancer burden by type III MC may be attributed to its low THC concentrations, rather than for its high CBD concentrations. Nevertheless, the THC:CBD ratio is not a good enough predictor for treatment response and other MC components (e.g., concentration of other phytocannabinoids or terpenoids) should be assessed and matched for some cancer symptoms. Hence, keeping in mind that we found no superior beneficial effects for type I MC treatments other than for sleep duration and due to the abuse potential of THC [52], type III MC treatments may be preferred for palliative oncology patients. Notably, previous clinical trials that compared cannabis-based medications, such as THC, CBD and equal THC:CBD products for chronic non-cancer pain patients, demonstrated superiority for equal THC:CBD products [53,54]. As whole-plant medical cannabis treatment is more complex than just its THC/CBD concentrations, with more than 500 components, of which over 100 are phytocannabinoids [20], the comparison to the relative inferiority of type II treatments in our study cannot be assessed.

This study has few limitations. First, no control or placebo groups were assigned. Hence, prudent interpretation of the results is needed. Second, self-report bias may have occurred. To diminish this bias, only validated questionnaires were utilized and patient responses were kept anonymous from their physician. Third, since we investigated palliative cancer patients, with a short life expectancy prognosis, a relatively short duration of follow-up was conducted. Future studies should attempt to extend the follow-up period. Fourth, the heterogeneity of our sample prevents us from making any generalization of our findings to a specific cancer etiology. Fifth, survival bias may have occurred, since patients that discontinued MC treatment, due to ineffectiveness or AEs and patients that passed away could not contribute their follow-up status. Fifth, selection bias might have also occurred, since extremely advanced patients might have not been included due to the study design. Lastly, we suggest repeating this study in a placebo-controlled randomized clinical trial, using standardized cannabis products with known phytocannabinoid and terpenoid composition.

## 4. Materials and Methods 

### 4.1. Study Design

This is an intermittent subgroup study of an ongoing, multi-center, prospective study that is being conducted since January 2019, and was analyzed at September 2020. The institutional Ethics Committees of the Haemek Medical Center, Israel (#0137–18-EMC) and of the Galil Medical Center, Israel (#0010–19-NHR) approved the study. This was a pure observational study with no interventional component whatsoever, so registration at the Clinical Trials Register was not required. Importantly, no recognizable information on participating patients is published in this article. 

Hebrew speaking patients aged ≥18 years licensed for the first time for MC for treating metastatic cancer pain and for chemotherapy-related nausea, vomiting and/or pain, were eligible for participation in the study. After explaining the study procedures, participating oncologists who regularly issue MC licenses on their own clinical discretion obtained written informed consents from eligible patients. Copies of the consent forms along with the patients’ cancer diagnoses, cancer treatment and contact information were sent to the study coordination center. To avoid any possible influence of collected data on physicians’ decisions regarding clinical management of their patients, prescribing physicians had no access to data collected on individual patients. 

Patients were instructed to complete the study questionnaires at baseline, before MC treatment initiation (T_0_; within few days from MC prescription), and at one (T_1_), three- and six-months following treatment initiation. In this subgroup analysis, we selected to present only the short-term (T_1_) effects of MC treatment. The baseline questionnaire consisted of 174 questions at baseline and a variable number of about 220 follow-up questions, which were presented in a dynamic format customized to individual responses where responses on a particular question determined the subsequent questions asked. In order to further reduce study burden, patients were also given the choice to skip questions. Hence, each patient completed a unique set of questions and each question received a different number of responses. Data was collected online by the secured survey technology Qualtrics^®^ (Provo, Utah, version 12018) [55]. Whenever patients had difficulties with the use of the web platform, the questionnaires could be completed by phone, with the assistance of the study coordinator. No financial compensation was offered to participating patients. The STROBE statement checklist for cohort studies is presented in the Supplementary Materials.

### 4.2. Study Questionnaires

Physician reported information included cancer diagnosis, classification of malignant tumors (TNM), cancer treatment protocol and the Eastern Cooperative Oncology Group (ECOG) Performance Status score. Patient reported information included demographics, analgesics consumption, MC treatment characteristics as well as Hebrew validated oncology-related questionnaires, including (1) the study’s primary outcome measure of “average weekly pain intensity” on a 0–10 numerical pain scale (NPS) and the weekly average worst and least pain intensities; (2) Memorial Symptom Assessment Scale (MSAS) [56]; (3) The short-form McGill Pain Questionnaire (SF-MPQ) [57]; (4) Pain Disability Index (PDI) [58]; (5) Quality of life—EuroQol (EQ5) [59]; (6) Pittsburgh Sleep Quality Index (PSQI) [60]; (7) Beck Depression Inventory II (BDI-II) [61]; (8) Pain Catastrophizing Scale (PCS) [62]; and (9) General Anxiety Disorder (GAD-7) [63]. Using a predetermined list [14], patients were requested to report adverse effects (AEs) at each follow-up time-point that they could attribute directly to MC treatment. AEs were later classified as serious or non-serious, according to the FDA definition [64].

### 4.3. Phytocannabinoids Dose Assessment

Since the Israeli MCU reform [65], MC cultivators in Israel are required to accurately indicate the THC and CBD concentrations in their products [33]. We calculated the monthly doses of THC and CBD only for patients that reported fully on their MC treatment regimen, according to the products that patients reported to consume, alongside with their total and product specific monthly doses. For example, if a patient reported on consuming 10 g of THC10/CBD10 product and another 10 g of THC20/CBD4 product, the patient calculated amount of monthly consumption is 6000 mg and 2800 mg of THC and CBD, respectively. Notably, MC cultivators in Israel are not required to report on terpenoids concentrations in their MC products.

### 4.4. Statistical Analysis

R software (V.1.1.463) with atable [66], ggstatsplot [67] and tidyverse [68] packages were used to analyze differences between three MC treatments: Type I (patients consuming THC-dominant cultivars only), type III (patients consuming CBD-dominant cultivars only; Type III) and type II (patients consuming either hybrid cultivars with similar THC:CBD ratio; Type II, or patients consuming a combination of THC-dominant and CBD-dominant cultivars in the same month) [34]. Chi square or Kruskal–Wallis rank tests were conducted to establish similarity of demographic data between the three treatments. The Shapiro-Wilk test of normality demonstrated non-normal distribution for all measures. Thus, data is presented as median (IQR, Q1-Q3, i.e., quartiles 25 and 75). Differences were considered significant at the *p* < 0.05 level. Incidences are presented as numbers and percentages of patients. Notably, due to the exploratory nature of the study and many potential subgroup analyses, no sample size was determined a priori.

### 4.5. Data Sharing

All data requests should be submitted to the corresponding author for consideration. Access to anonymized data may be granted following review of the request.

## 5. Conclusions

In conclusion, this prospective, short-term cohort of palliative cancer patients demonstrated an overall mild improvement of most investigated parameters, regardless of its phytocannabinoid dominance. This is the first study that investigated the variability between three different classes of regulated and accurately labelled MC products, using precise doses of both phytocannabinoids, THC and CBD. Keeping in mind our relatively small sample size, short-term follow-up and lack of control and placebo groups, we could not elucidate any differences in beneficial effects between MC treatments for most outcomes, but we were able to demonstrate some differential effects between them. Thus, we can cautiously recommend type III treatments and not type I or type II treatments be prescribed for oncology patients with a high burden of physical cancer symptoms. However, if the patient’s main complaint is short sleep duration, type I treatments are preferred. Future studies should further investigate the role of different MC treatments in order to better elucidate our understanding of MC treatment complexities.

## Figures and Tables

**Table 1:** Demographic characteristics.

Parameters	Type I *n* = 56	Type III *n* = 19	Type II *n* = 33	Total *n* = 108	(χ^2^) ^†^/Kruskal–Wallis rank ^††^ (*p*-value) ^#^
**Median (IQR)**					
Age (years)	62 (49–68)	66 (54–74)	66 (56–72)	64 (52–72)	3.37 ^††^ (0.19)
Unknown	4	2	2	8	
**No. of patients (%)**					
Gender				Gender	
Male	25 (45)	6 (32)	15 (45)	Male	25 (45)
Female	31 (55)	13 (68)	18 (55)	Female	31 (55)
**Median (IQR)**					
Weight (kg)	72 (65–80)	69 (53–77)	66 (55–80)	70 (59–80)	2.12 ^††^ (0.35)
Unknown	7	5	5	17	
BMI	25 (22–29)	24 (21–26)	26 (21–28)	25 (22–28)	0.70 ^††^ (0.70)
Unknown	12	7	6	25	
**No. of patients (%)**					
Comorbidities (yes)	19 (34)	5 (26)	7 (21)	31 (29)	1.21 ^†^ (0.54)
Unknown	1	1	3	5	
Past cannabis experience (yes)	17 (30)	5 (26)	7 (21)	29 (27)	0.88 ^†^ (0.64)

^†^, Pearson’s Chi-squared test; ^††^, Kruskal–Wallis rank sum test; kg, kilograms; BMI, body mass index; IQR, inter quartile range; *n*, number of patients; where missing *n* is not specified, there are no missing data; Type I, THC-dominant treatments; Type III, CBD-dominant treatments; Type II, equal THC:CBD concentration treatments; ^#^, Significance between the three different treatment regimens.

**Table 2:** Cancer characteristics.

Parameters	Type I *n* = 56	Type III *n =* 19	Type II *n* = 33	Total *n* = 108	χ^2^ (*p*-Value) ^#^
**No. of patients (%)**					
**Solid tumor etiology**					
Breast	19 (34)	3 (16)	8 (24)	30 (28)	10.81 (0.21)
Lung	9 (16)	2 (11)	4 (12)	15 (14)	
Colon	8 (14)	5 (26)	2 (6)	15 (14)	
Ovaries	1 (2)	2 (11)	2 (6)	5 (5)	
Other	18 (32)	6 (32)	17 (52)	41 (38)	
**Solid tumor staging**					
I	3 (5)	1 (5)	3 (9)	7 (7)	4.30 (0.64)
II	8 (14)	1 (5)	6 (18)	15 (14)	
III	4 (7)	3 (16)	3 (9)	10 (9)	
IV	26 (46)	11 (58)	11 (33)	48 (44)	
Unknown	15	3	10	28	
**Oncological treatment line**					
1st	30 (54)	8 (42)	19 (58)	57 (53)	2.65 (0.26)
≥2nd	18 (32)	10 (53)	9 (27)	37 (34)	
Unknown	8	1	5	14	
**Oncological treatment ^†^**					
Chemotherapy	27 (48)	11 (58)	18 (55)	56 (52)	0.67 (0.72)
Biological	8 (14)	3 (16)	4 (12)	15 (14)	0.15 (0.93)
Hormonal	7 (12)	0	4 (12)	11 (10)	2.61 (0.27)
Immunological	6 (11)	1 (5)	3 (9)	10 (9)	0.50 (0.78)
Radiation	1 (2)	1 (5)	0	2 (2)	1.84 (0.40)
**ECOG score**					
≤1	39 (70)	12 (63)	25 (75)	76 (70)	0.15 (0.92)
≥2	14 (25)	5 (26)	8 (24)	27 (25)	
Unknown	3	2	0	5	

χ^2^, Pearson’s Chi-squared test; *n*, Number of patients; ECOG, Eastern Cooperative Oncology Group Performance Status; ^†^, numbers does not add up to 100% due to concomitant treatments; where missing *n* is not specified, there are no missing data; ^#^, Significance between the three different treatment regimens; Type I, THC-dominant treatments; Type III, CBD-dominant treatments; Type II, equal THC: CBD concentration treatments.

**Table 3:** Medical cannabis treatment regimen-related adverse effects.

Parameters	Type I *n* = 56	Type III *n* = 19	Type II *n* = 33	χ^2^ (*p*-Value) ^#^
**No. of patients (%)**				
Overall adverse effects	10 (18)	3 (16)	11 (33)	3.02 (0.22)
Central nervous system	6 (11)	1 (5)	7 (21)	2.94 (0.23)
Gastrointestinal	3 (5)	2 (11)	4 (12)	1.30 (0.52)
Psychological	2 (4)	1 (5)	4 (12)	2.37 (0.30)
Musculoskeletal	1 (2)	0	3 (9)	3.78 (0.15)
Ophthalmic	1 (2)	1 (5)	2 (6)	1.17 (0.56)
Cardiovascular	0	0	2 (6)	4.44 (0.11)
Auditory	0	0	2 (6)	4.44 (0.11)

χ^2^, Pearson’s Chi-squared test; AEs, adverse effects; ^#^, significance between the three different treatment regimens; Type I, THC-dominant treatments; Type III, CBD-dominant treatments; Type II, equal THC:CBD concentration treatments.

**Table 4:** Reported non-serious, MC-related adverse events.

**Total *n* = 108**	
**Central nervous system**	**No. of patients (%)**
Confusion	4 (4)
Disorientation	4 (4)
Impaired attention	3 (3)
Dizziness	9 (8)
Falls	3 (3)
Feeling drunk	6 (6)
Decreased physical sensation	4 (4)
Impaired balance	6 (6)
Impaired memory	5 (5)
Impaired psychomotor functions	5 (5)
Impaired coordination	5 (5)
Increased awareness	5 (5)
Impaired speech	5 (5)
Tiredness	9 (8)
Vertigo	5 (5)
**Gastrointestinal**	**No. of patients (%)**
Abdominal discomfort	7 (7)
Abdominal pain	6 (6)
Decreased appetite	7 (7)
Increased appetite	3 (3)
Loss of appetite	5 (5)
Bad taste	8 (7)
Constipation	5 (5)
Diarrhea	6 (6)
Dry mouth	6 (6)
Heartburn	5 (5)
Decreased mouth sensation	5 (5)
Mouth ulcers	5 (5)
Nausea	6 (6)
Vomiting	6 (6)
Mouth pain	5 (5)
Thirst	6 (6)
**Psychological**	**No. of patients (%)**
Unusual thinking	4 (4)
Anxiety	4 (4)
Bad mood	6 (6)
Sweet craving	5 (5)
Depression	4 (4)
Decreased interest	5 (5)
Euphoria	4 (4)
Forgetfulness	4 (4)
Hallucinations	4 (4)
Hyperactivity	3 (3)
Loss of time sensation	4 (4)
Nervousness	4 (4)
Nightmares	3 (3)
Paranoia	3 (3)
Weird dreams	2 (2)
Psychosis *	2 (2)
**Musculoskeletal**	**No. of patients (%)**
Bone pain	3 (3)
Joint pain	3 (3)
Jaw stiffness	2 (2)
Decreased motor ability	2 (2)
Limb weakness	3 (3)
Muscle pain	3 (3)
Tremor	1 (<1)
**Cardiovascular**	**No. of patients (%)**
Hypertension	1 (<1)
Hypotension	2 (2)
Irregular pulse	1 (<1)
Orthostatic hypotension	2 (2)
Palpitations	2 (2)
**Ophthalmic**	**No. of patients (%)**
Blurred vision	4 (4)
Red eyes	1 (<1)
Vision alterations	1 (<1)
Itchy Eyes	1 (<1)
Light sensitivity	3 (3)
**Auditory**	**No. of patients (%)**
Ears buzzing	2 (2)
Decreased hearing	2 (2)
Noise sensitivity	1 (<1)

* diagnosed by psychiatrist; some of the adverse effect reports are concomitant.

**Table 5:** Difference from baseline in outcome parameters.

Parameters	T_0_	T_1_	*n* = 108	Two-Sample Kolmogorov-Smirnov Test (*p*-Value)	Decrease in Score Indicates
	**Median (IQR)**		**Unknown**		
Weight (Kg)	72 (60–80)	70 (59–80)	17	0.10 (0.72)	Worsening
BMI (weight (kg)/[height (m)]^2^)	26 (22–29)	25 (22–28)	25	0.07 (0.97)	Worsening
Weekly least pain intensity (NPS, 0–10)	5 (2–7.2)	3 (1–6)	35	0.16 (0.27)	Improvement
Weekly worst pain intensity (NPS, 0–10)	8 (6–9)	6 (5–8)	35	0.20 (0.10)	Improvement
Weekly average pain intensity (NPS, 0–10)	7 (3–8.8)	5 (1.5–7)	5	0.22 (<0.05)	Improvement
Affective pain intensity (McGill questionnaire, 0–12)	7 (4.5–9)	4 (2–7)	43	0.34 (<0.01)	Improvement
Sensory pain intensity (McGill questionnaire, 0–33)	18 (14–24)	13 (7–20)	45	0.28 (<0.05)	Improvement
Pain catastrophizing scale (PCS, 0–52)	28 (15–37)	22 (7–36)	17	0.13 (0.47)	Improvement
Sleep quality (PSQI global score, 0–21)	12 (9–15)	9 (5.2–12)	30	0.29 (<0.01)	Improvement
Sleep duration (h)	5 (4–6.5)	6 (5–7.5)	8	0.25 (<0.05)	Worsening
Sleep latency (min)	45 (30–60)	30 (15–60)	11	0.17 (0.10)	Improvement
Depression (BDI, 0–63)	17 (10–24)	15 (9–21)	10	0.10 (0.64)	Improvement
Quality of life (EQ-5, 0–10)	4 (3–6)	3 (2–5)	5	0.13 (0.37)	Improvement
Anxiety (GAD-7, 0–21)	8 (2.8–14)	5 (2–11)	8	0.14 (0.28)	Improvement
MSAS distress index (0–100)	44 (25–64)	34 (19–46)	0	0.20 (<0.05)	Improvement
MSAS physical index (0–120)	38 (18–50)	27 (12–40)	0	0.23 (<0.01)	Improvement
MSAS psychological index (0–60)	24 (14–40)	16 (8–32)	0	0.22 (<0.05)	Improvement
	**No. of patients (%)**				
Analgesics consumption (yes)	60 (56)	40 (37)	1	6.8 (<0.01)	Improvement

BL, Baseline; m, meter; NPS, Numerical pain scale; PSQI, Pittsburgh Sleep Quality Index; BDI, Beck depression index; EQ-5, EuroQol questionnaire; GAD-7, General anxiety disorder questionnaire; IQR, Inter quartile range; Kg, Kilograms; BMI, Body mass index; MSAS, Memorial Symptom Assessment Scale; *n*, Number of patients; Range is indicated next to each parameter.

## Supplementary Materials

The following are available online at https://www.mdpi.com/1424-8247/13/12/435/s1, Methods S1: STORBE checklist.
